# Changes in the thermodynamical profiles of the subsurface ocean and atmosphere induce cyclones to congregate over the Eastern Arabian Sea

**DOI:** 10.1038/s41598-023-42642-9

**Published:** 2023-09-22

**Authors:** C. S. Abhiram Nirmal, S. Abhilash, Max Martin, Syam Sankar, M. Mohapatra, A. K. Sahai

**Affiliations:** 1 Advanced Centre for Atmospheric Radar Research (ACARR), Cochin University of Science and Technology (CUSAT), Kochi, 682022 India; 2 Department of Atmospheric Sciences, Cochin University of Science and Technology, Kochi, 682016 India; 3 Department of Anthropology, University of Sussex, Brighton, UK; 4 National Centre for Medium Range Weather Forecasting (NCMRWF), Sector-62, Noida, Uttar Pradesh 201309 India; 5 India Meteorological Department (IMD), New Delhi, 110003 India; 6 Indian Institute of Tropical Meteorology (IITM), Pune, 411008 India

**Keywords:** Climate change, Attribution, Atmospheric dynamics, Climate sciences

## Abstract

The Arabian Sea accounts for a small fraction of Tropical Cyclones—about 2% of the annual global mean. However, the damage they might inflict there and along its coastlines, which are thickly populated, is considerable. This study explores the influence of the changes in the vertical profiles of atmosphere and oceanic environment throughout the seasons of March–June (MAMJ) and October–December (OND) in clustering the cyclogenesis over the Eastern Arabian Sea (EAS) next to the Indian West coast in recent decades. Further investigation has been done into the precise contribution of atmospheric and oceanic factors to fluctuations in cyclone intensity throughout the MAMJ and OND seasons separately. Two seasons have been studied independently in order to better understand the distinct influences of the vertical fluctuation of atmospheric factors and the thermal structure of the oceanic subsurface on cyclogenesis. More severe cyclones are caused by high tropical cyclone heat potential, and ocean subsurface warming present in this sea region influences the genesis of storms mostly during MAMJ. On the other hand, mid tropospheric relative humidity and thermal instability influences more on increasing cyclogenesis and its clustering over EAS during OND season. The findings suggest that large-scale oceanic subsurface conditions have a crucial influence on cyclogenesis over EAS through oceanic sensitivity to atmospheric forcing. This cyclone tendency and its clustering over EAS needs attention in terms of forecasting, catastrophe risk reduction, and climate change adaptation due to the security of coastal urban and rural habitats, livelihoods, and essential infrastructure along the coasts.

## Introduction

A recent increase in the North Indian Ocean (NIO) Tropical Cyclone (TC) trends poses challenges in forecasting, disaster risk reduction and humanitarian interventions on the densely populated coasts on the ocean rim. As severe cyclonic storms and associated compound hazards are increasing along the west coast of India, it is important to understand regional scale driving mechanisms in devising local adaptation.

This paper looks at changes in TC spatial patterns, frequency, and intensity over the Arabian Sea during the pre- and post-monsoon seasons. It considers local Ocean Heat Content (OHC, denoting temperature change, density of seawater and specific heat capacity from the surface to deep ocean) as a driver of Tropical Cyclone Heat Potential (TCHP, excess heat in the ocean over the 26$^{\circ }$ isotherm) indicating the ocean thermal profile conducive for TC formation^1–3^. Most studies related to TCs mainly consider the Sea Surface Temperature (SST) to represent the ocean state^4–10^ while the subsurface temperatures of NIO has been found not to co-vary with the surface temperatures^11,12^. Several studies have reported that TCs interact and intensify after encountering regions of higher TCHP values^13–18^. One recent example of such a case in the NIO is that of Very Severe Cyclonic Storm (VSCS) Ockhi (2017) that originated in the Bay of Bengal (BoB)^19^. During its development stage, cyclone Ockhi encountered anomalously high SST and ocean subsurface temperatures, leading to it’s rapid intensification^18^. This emphasises the importance of the subsurface ocean temperature, OHC and, by extension of TCHP, to the study of TCs. In light of this, the relative role of each individual potential parameters and TCHP is examined with respect to the changes in TC frequency and intensity over NIO.

NIO has witnessed five TCs per year with one to two events in the Arabian Sea (AS-west of ${77.5}^{\circ }\,\hbox {E}$) a decade ago, while the global annual mean remained 85 TCs^20,21^. In the AS, TCs are more prevalent during March-June (MAMJ) marking the pre-monsoon (and early monsoon) (MAMJ) and October–December (OND) post-monsoon seasons^20,22^. Amidst increasing TC activity, post-monsoon events are gaining intensity^6,23^. The first recorded post-monsoon Extremely Severe Cyclonic Storm (ESCS, maximum winds above ${46} \hbox {m s}^{-1}$ or ${168}\,\hbox {km h}^{-1}$) of the AS occurred in October 2014 (Cyclone Nilofar, ${56.6}\,\hbox {m s}^{-1}$ maximum wind speed)^24^, followed by two back-to-back ESCSs during the next post-monsoon season (2015) (TCs Chapala and Megh)^25,26^. Among the five events in 2019, ESCS Maha coexisted briefly with the Super Cyclonic Storm (SuCS-${222}\,\hbox {km h}^{-1}$ and above) Kyar as an unprecedented double event in the satellite era of 1961–2018^6,27,28^. Meanwhile, the total duration of Very Severe Cyclonic Storms (VSCS, ${119}\!-\!\!{221}\hbox { km h}^{-1}$) has also increased three-fold and Cyclonic Storms (CS-${62}\!-\!\!{88}\hbox {km h}^{-1}$) by 80%^28^.

There have been unusual tracks and intensification of storms. For instance, Cyclone Ockhi formed in the southwestern Bay of Bengal on 28 Nov 2017, traversed 2500 km over the Indian Ocean (IO) and the Arabain Sea (AS), rapidly intensifying into a VSCS near the coasts of Sri Lanka, Tamil Nadu, and Kerala^29^. These changes in storm patterns have been attributed to various climatic and environmental factors. Increase in post-monsoon TC frequency has been attributed to rising aerosol and black carbon emissions reducing the vertical shear of horizontal wind caused by the tropical easterly wind in the upper troposphere and the westerly jet in the lower troposphere^23,30^. In a warming globe, an anomalous increase in Potential intensity (PI, the maximum intensity for observed storms) and OHC in May and weakening of the summer monsoon circulation have been shown to drive a trend of more ESCS in the AS^6,30,31^. The recent warming trend of the IO^32–34^ is most pronounced in its western part^35,36^, where the mean summer Sea Surface Temperature (SST) has increased from 26.5$^{\circ }$ to 28$^{\circ }$ over the past century^35^. The Indo-Pacific Warm Pool (IPWP), a region where SST remain above 28$^{\circ }$ round the year, has been warming and expanding over the past century. Over the Indian Ocean region, it has doubled in aerial extent as compared to its size a century back^32,37,38^. As such, the world oceans were the warmest in 2021^39^.

This warming is linked to Earth Energy Imbalance (EEI)—the difference between emitted and reflected solar radiation—that has doubled worldwide between 2005 and 2019^26,39^. EEI increases OHC as over 90% of EEI is stored in the ocean, with deep seas increasingly determining the energy budget. The total increase in the OHC of upper 2000 m during 1998–2015 is estimated at ${15.2 \times 22}$ J, of which the IO accounts for 24%^26,39^. In the IO, the abrupt OHC increase accounting for over 70% of the global ocean heat gain in the upper 700 m of the ocean since 2003 has been attributed to increased heat transport from the Pacific Ocean to the Indian Ocean by the Indonesian throughflow (ITF) current^40^. In this background of general heat gain in the IO, this study looks at its implications for the Eastern AS (EAS).

The majority of earlier research has generally focused on the overall rising trends in severe cyclonic storms across the whole Arabian Sea basin. The causes of the increased cyclogenesis potential over the Eastern Arabian Sea, however, are the focus of this study. This study also examines how multiple meteorological and oceanic factors contribute to the genesis of cyclones in the Eastern Arabian Sea, as well as how these factors differ and interact with one another during the MAMJ and OND seasons. Additionally, earlier research has included either the spatial trend or the area averaged trend of vertically integrated factors. In order to comprehend the changes in the atmosphere and ocean brought on by climate change and leading to the observed changes in the cyclogenesis over the Eastern Arabian Sea, the vertical variation of the trend at various height levels in the atmosphere and depth levels in the oceans has for the first time been analysed in this study.

## Results

### Changes in the tropical cyclone activity over Arabian Sea

**Figure 1:**
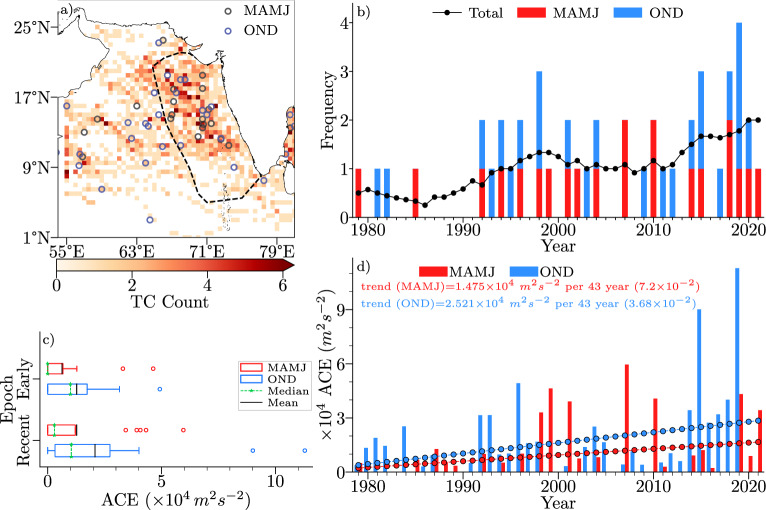
(**a**) Cyclogenesis locations during 1979–2021 period overlaid on spatial count of TCs binned to a ${.5}^{\circ }\times {.5}^{\circ }$ grid (**b**) TC frequency with an 11-years running mean of both MAMJ and OND seasons (**c**) distribution of seasonal ACE for the two epochs and (**d**) time-series of ACE for both seasons of TCs formed east of ${64}^{\circ }\,\hbox {E}$.

Cyclone track density and cyclogenesis location during MAMJ and OND season for the period from 1979 TO 2021 are presented in Fig. 1a to highlight the region of interest over EAS, where most of the cyclogenesis locations and tracks are clustered. This trend implies that the Arabian Sea is witnessing an increasing number of systems developing into Tropical Cyclone (TC)-intensity in the recent epoch, pointing to a change in the environmental factors conducive for TC activity.

Figure 1b shows the frequency of TCs in the AS for MAMJ and OND seasons by considering only those systems having a Maximum Sustained Wind (MSW) speed of more than 34 knots as a single category. The frequency of TC, as depicted by the 11-years running mean, reveals that systems are increasing over AS. These changes imply that the Arabian Sea is witnessing an increasing number of systems developing into TC-intensity in the recent epoch, pointing to a change in the environmental factors conducive to TC activity.

Figure 1c shows the total seasonal ACE time-series in the western Arabian Sea while Fig. 1d depicts the distribution of seasonal ACE for two epochs—early (1979–2000) and recent (2001–2021). Seasonal ACE has increased by $1.475\times 10^{4}\,\hbox {m}^2\,\hbox {s}^{-2}$ and $3.68\times 10^{4}\,\hbox {m}^2\,\hbox {s}^{-2}$ during the analysis period for MAMJ and OND, respectively (significant at 90% confidence interval). The distribution of seasonal ACE for both epochs is shown in Fig. 1d with the most substantial rise occurring during MAMJ. More TCs with higher ACEs are observed in the recent epoch during both seasons.

### Changes in the atmospheric conditioning factors

We have taken most of the cyclogenesis parameters from^41–43^ that have been used for developing Genesis Potential Parameter (GPP) for different ocean basins^44,45^. Accordingly, these parameters have been considered in this study and most important parameters, which exhibit significant increasing trend over EAS.

**Figure 2:**
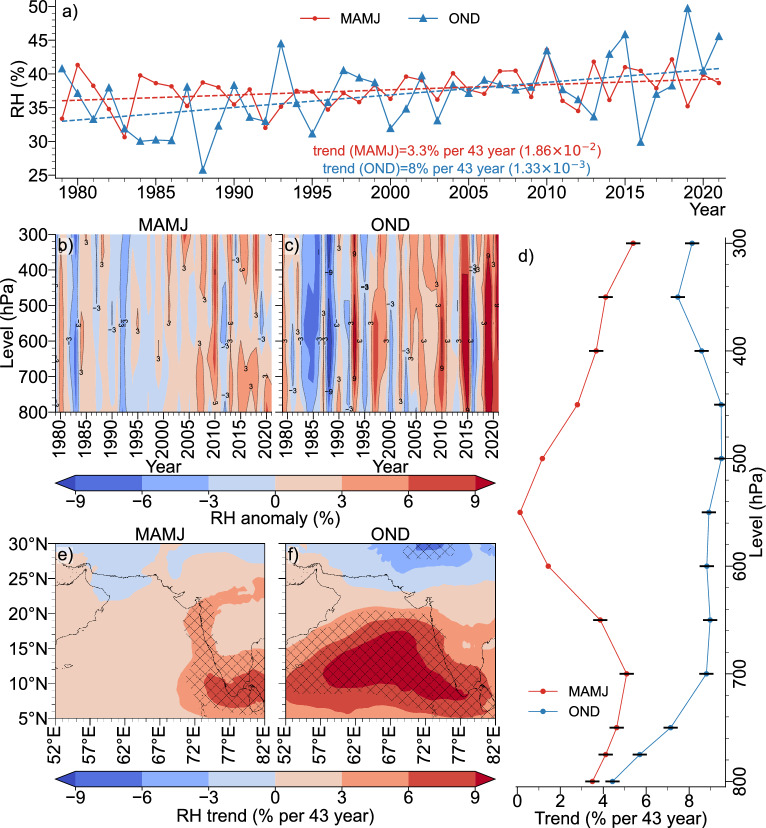
(**a**) Area and vertically averaged mid-tropospheric RH time-series (**b**) vertical profile time-series of area averaged RH for MAMJ (**c**)  Same as b, but for OND (**d**) Vertical profile of area averaged RH trend at each level (black markers indicate trend values significant at 95% confidence level (**e**) spatial trend of MRH for MAMJ ((the hatches indicate statistically significant areas at 95% confidence level) and (**f**) same as (**e**), but for OND.

The Mid-tropospheric RH (MRH) is increasing during both MAMJ and OND, with OND indicating a larger positive trend than MAMJ, significant at 95% confidence level (Fig. 2a). This increasing trend is evident in the area-averaged vertical anomaly plots (Fig. 2b,c) as well as in the vertical profile of trend values at each level (Fig. 2d). Fig. 2e,f show the spatial trend of MRH over the AS. A robust positive trend is observed over most of the AS during OND with values above 9% observed over a significant portion of the AS (Fig. 2f). Although the MAMJ season exhibit an overall positive trend in the AS (Fig. 2e), the trend is comparatively lower than OND. As indicated by Fig. 2d, the increasing trend in MRH is not confined to a single level; instead, it is observed throughout the mid-troposphere, with the post-monsoon season having a higher trend at all levels than the pre-monsoon season. Therefore, the upward trend in cyclones during OND season, as compared to MAMJ season, is primarily influenced by the increase in MRH.

**Figure 3:**
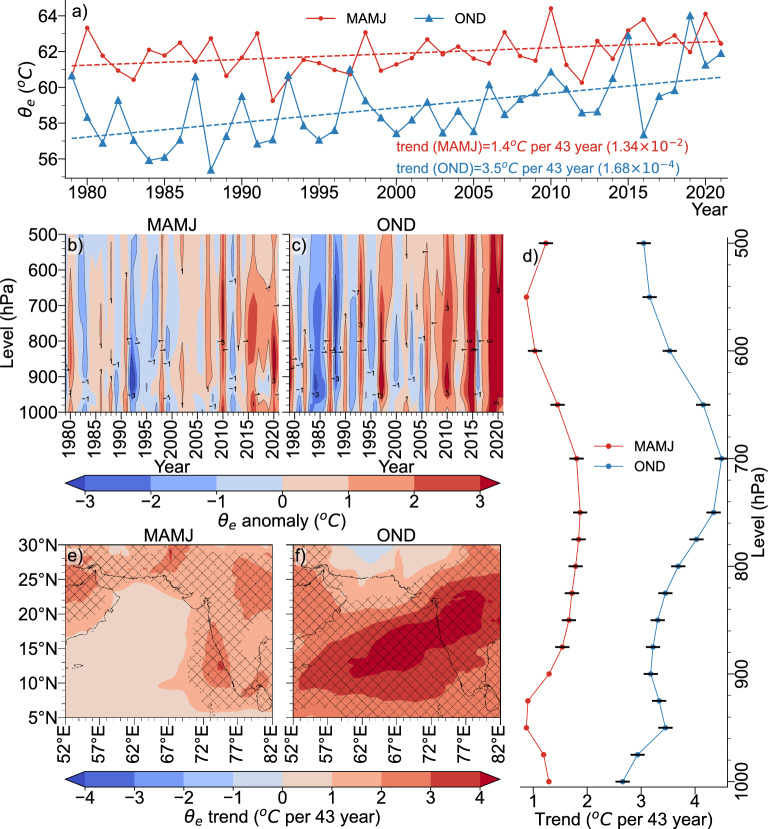
(**a**) Area and vertically averaged $\theta _e$ time-series (**b**) vertical profile time-series of area averaged $\theta _e$ for MAMJ (**c**)  Same as (**b**), but for OND (**d**) Vertical profile of area averaged $\theta _e$ trend at each level (black markers indicate trend values significant at 95% confidence level (**e**) spatial trend of $\theta _e$ averaged between 300 and ${800}\hbox { hPa}$ for MAMJ (the hatches indicate statistically significant areas at 95% confidence level) and (**f**) same as (**e**), but for OND.

Higher equivalent potential temperature ($\theta _e$) values near the ocean surface is a manifestation of higher SSTs that enhance convection and moisture transport^46^. On the other hand, an increase in $\theta _e$ at 700 hPa is considered a crucial component of rapid intensification and associated reduction in central pressure of tropical storms^47^ and have been used for operational forecasting of cyclone intensification^48^. Hence, $\theta _e$ in both lower and middle troposphere is equally important in determining the thermal instability profile. Deshpande et al.^28^ also reported that, an increase in column averaged (950–150$\hbox { hPa}$) MSE, which is a reflection of the thermal instability, that is playing a dominant role in the observed increase in TC frequency in AS. Following^49^, we have considered mean $\theta _e$ between 1000 and ${500} \hbox { hPa}$ as a measure of thermal instability since $\theta _e$ at 1000 hPa include the effect of SST and heat fluxes and the mid-tropospheric $\theta _e$ include the effect of latent heat release associated with conditional instabilities and feedbacks during the storm intensification.

The area-averaged $\theta _e$ between 500 and ${1000}\hbox { hPa}$ shows an increasing trend during both MAMJ and OND seasons, as shown in Fig. 3a. The higher trend values in $\theta _e$ during OND season is mostly evident in recent decades, and the increase in middle-level $\theta _e$ might have been mostly contributed from the increase in middle-level humidity as discussed earlier. The vertical trend profile for $\theta _e$ (Fig. 3d) indicates that OND season has a higher trend throughout the mid-troposphere than MAMJ. The increased trend throughout the lower and mid-troposphere is supported by vertical anomaly plots (Fig. 3b,c), where the OND season show a substantial increase in $\theta _e$ during recent decade (Fig. 3c). The increased trend during OND is also evident in the spatial trend maps in the central and eastern Arabian Sea (Fig. 3e, f).

We found that there is no significant trend in the dynamical parameters like 850 hPa relative vorticity and vertical wind shear during both the seasons in agreement with the findings of^28^. Furthermore^50^, showed that the thermodynamic characteristics of the atmosphere and upper ocean are primarily responsible for controlling the evolution of storms. Hence we assume that both of these parameters are less influential in the observed changes and are not discussed in detail.

### Changes in the oceanic subsurface conditions

Sea-surface temperature (SST), a significant element in the air-sea interaction, has received a lot of interest in TC research. The ocean subsurface has, however, received much less attention, particularly when describing the regional variability of the cyclogenesis over the Arabian Sea, because the majority of current TC research studies pay close attention exclusively to the SST and upper ocean heat content to characterise the contribution of the ocean.

**Figure 4:**
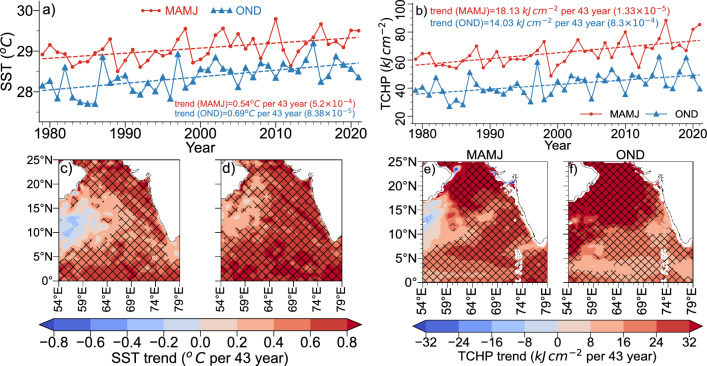
(**a**) Time-series of area averaged SST (**b**) same as (**a**), but for TCHP (**c**, **d**) spatial trend of SST for MAMJ & OND, respectively (hatching indicates statically significant areas at 95% confidence level) (**e**, **f**) same as (**c**, **d**), but for TCHP.

**Figure 5:**
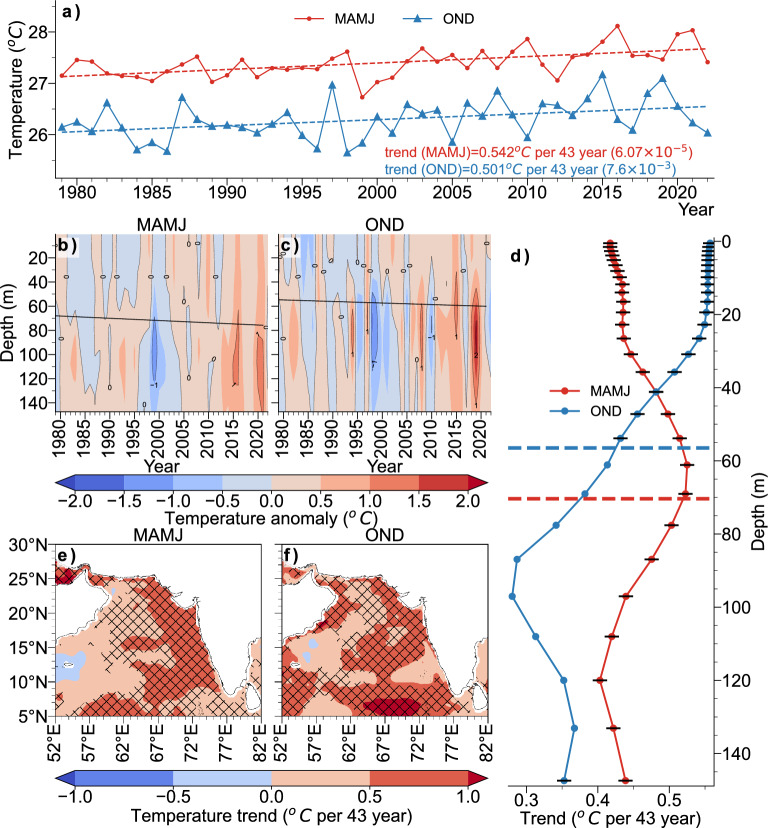
(**a**) Area and depth averaged ocean subsurface temperature time-series (**b**, **c**) area-averaged sub-surface temperature anomaly profile (black-line represents the 26$^{\circ }$ isotherm depth trend, for MAMJ and OND, respectively) (**d**) depth-profile of temperature trend at each level (black markers indicate levels with 95% confidence level, horizontal red and blue lines represent the climatological depth of 26$^{\circ }$ isotherm for MAMJ and OND, respectively) (**e**) spatial trend of ocean sub-surface temperature averaged between 0 and ${150}\hbox { m}$ for MAMJ (the hatches indicate statistically significant areas at 95% confidence level) and (**f**) same as e, but for OND.

Spatial trend analysis of SST revealed a warming trend all over the Arabian Sea during both MAMJ and OND, except for a small region over west-central AS during MAMJ (Fig. 4c,d). The area-averaged SST too shows a similar result, with OND season showing a higher positive trend than MAMJ (Fig. 4a). Hence, this basin wide warming trend not sufficient to explain the rigorous variability in the increase in cyclone frequency and clustering of cyclogenesis locations over EAS. TCHP is higher during MAMJ compared to OND in the AS (Supplimentary Fig. 3). Analysis showed that the TCHP over EAS has been increasing, with the area-averaged time-series showing trend values of ${18.13}\,\hbox {kJ cm}^{-2}$ and ${14.03}\,\hbox {kJ cm}^{-2}$, per 43 years, during MAMJ and OND, respectively (Fig. 4b). TCHP has been increasing throughout the AS except for some regions over western Arabian Sea (Fig. 4e,f), with the most increase observed over the northern AS during MAMJ and OND. This increase in the TCHP is consistent with the AS sub-surface warming and the deepening of 26$^{\circ }$ isotherm as observed in Fig. 5b,c, with both parameters contributing positively to increase the TCHP. The increase in frequency and intensity of TC could also be attributed to changes in subsurface water temperature, which controls the ocean’s vertical thermal structure. A study on the interaction between the ocean subsurface and TC activities is of major interest because of the strong influence that the ocean subsurface has on the energy transfer during the genesis and intensification of TC. Many studies have shown that the energy supply for a TC is significantly impacted by the upper subsurface, which extends to depths of 100–${150}\hbox { m}$^50–52^ and the temperature of this layer over the Arabian Sea varies significantly on the inter annual time scale.

Area and depth averaged Ocean subsurface temperature from 0 to ${150}\hbox { m}$ given in Fig. 5a shows the increasing ocean subsurface temperature of the EAS. The sub-surface temperature has increased by more than 0.5$^{\circ }$ in 43 years (1979–2021) during both seasons. Depth anomaly-profiles (Fig. 5b,c) show the extent of sub-surface warming, indicating warming throughout the depth 0–150$\hbox { m}$. The straight, black lines in Fig. 5b,c denote the 26$^{\circ }$ isotherm depth trend. The MAMJ season has a deeper 26$^{\circ }$ isotherm depth compared to OND. Fig. 5b,c indicate that the 26$^{\circ }$ isotherm is deepening. Deepening of the 26$^{\circ }$ isotherm is particularly concerning as it will contribute to higher TCHP, positively affecting the genesis and intensification of TCs. Our analysis found that the 26$^{\circ }$ isotherm deepens 8 m per 43-years during MAMJ and 5.5 m per 43-years during OND.

Figure 5d shows the trend of sub-surface temperature at each depth level, with the black markers representing 95% confidence level. The horizontal dashed lines represent the climatological depth of 26$^{\circ }$ isotherm, showing that it is deeper during MAMJ than OND. The OND trend is higher than that of MAMJ above 50 m, below which MAMJ shows a higher trend than OND. The trend during OND decreases as the depth increases and is over 0.5$^{\circ }$ per 43-years above 50 m. Spatial trend of area-averaged subsurface averaged between 0 and ${100}\hbox { m}$ warming all over the ocean (Fig. 5e,f). Higher trend is observed to the western coast of Indian peninsula during MAMJ, and in the central and southern AS during OND (Fig. 5e,f).

Essentially, these findings indicate that the AS sub-surface temperature is on the rise. This is in line with the findings of Albert et al.^3^, where they attribute the AS sub-surface warming during OND to the increase in the intensity of the second downwelling Kelvin wave originating in the Equatorial Indian Ocean during October. On the other hand, during pre-monsoon, surface heat flux exhibited an increasing trend since 2000, while vertical entrainment showed a decreasing trend^3^. This found that the depth of 26$^{\circ }$ isotherm increased as deep as 80 m during MAMJ and the increasing trend in sub-surface water is more pronounced below 50 m depth. This suggests that the warm water volume increased significantly in MAMJ season and can be considered as a more influential oceanic parameter for the observed increase in cyclone count and intensity during MAMJ season.

Large-scale upper-ocean temperature stratification seems to play a significant role in cyclogenesis across the Eastern Arabian Sea (EAS) on seasonal time scale. In line with the past observational studies^53–55^, we assume that this oceanic dependence, in addition to the influence of changes in atmospheric state, may influence the genesis of intense cyclones in a changing climate conditions.

This analysis reveals that, besides SST and ocean heat content in top layers, subsurface temperature profile below 26$^{\circ }$ isotherm has also been suggested to be potentially influential for TC genesis. Under global warming, the subsurface vertical temperature profile can be sharpened in most of the cyclogenesis regions of Arabian sea, which may contribute to a stronger ocean coupling effect during the intensification of future TCs. The climate variability modes such as, ENSO and IOD can also modulate the number of TCs by modulating the ocean and atmosphere states. The ENSO and IOD impact on TC genesis provides a cautionary tale for a potentially complementing and sometimes offsetting role of the dynamic and thermodynamic impacts on cyclogenesis due to a coupling between SSTs, surface winds, and the thermocline by altering the subsurface thermal stratification of the ocean.

This finding suggests that since large-scale oceanic subsurface conditions regulate oceanic sensitivity to atmospheric forcing, they play a crucial role in regulating tropical cyclone intensity. As a result, we need to comprehend how the oceans affect cyclone intensity, as well as their influence and magnitude in relation to alterations in atmospheric conditions. Additional evidence for this relationship is provided by ocean subsurface thermal measurements and can be used as potential predictor for seasonal cyclogenesis over EAS basin.

### Genesis potential index (GPI) and maximum potential intensity

By using the TC Genesis Potential Index (GPI), the influence of the large-scale environmental factors on TC genesis is examined. Thermodynamic state of the atmosphere and the ocean subsurface temperature profile over Eastern Arabian Sea and its link with seasonal GPI has been investigated. The linkage between ocean subsurface and other oceanic or atmospheric parameters which have influence on the cyclogenesis and evolution has been analysed with the help of commonly used GPP index for seasonal and climate change studies^44^. Moreover, the depth of 26$^{\circ }$ isotherm is deeper during MAMJ than in OND seasons (Fig. 5b–d). The subsurface ocean environments is not spatially uniform and the coupling between the sub-surface ocean and atmosphere above may be distinct over different regions within Arabian Sea basin. Therefore, the relationship between ocean subsurface temperature and the TC activities over the Arabian Sea basin may vary between seasons. The linkage between ocean subsurface and other oceanic or atmospheric parameters which have influence on the cyclogenesis and development has been analysed with the help of commonly used GPI as well as by modifying the GPI following^56^. However, in order to account the spatial variability in the subsurface temperature and TCHP, spatially varying threshold have been used to modify GPI in the present work.

**Figure 6:**
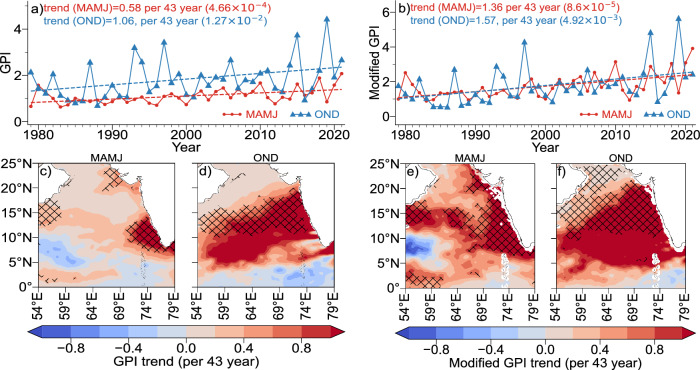
(**a**) Area-averaged time-series of original GPI^44^ (**c**, **d**) spatial trend of GPI, for MAMJ and OND, respectively (the hatches indicate statistically significant areas at 95% confidence level) (**b**) same as a, but for modified GPI (**e**) same as (**c**), but for modified GPI and (**f**) same as (**d**), but for modified GPI.

To better reflect the increasing trend of RH in the mid-troposphere, we used RH averaged over the 850–${400}\hbox { hPa}$ levels for calculating the GPI rather than using RH at the 600 hPa level as in^44^. Fig. 6a,c,d show the analysis of original GPI^44^ while Fig. 6b,e,f show the modified GPI. Fig. 6a shows that GPI is higher during OND than MAMJ, and shows a higher trend. In the most recent period, positive trends have been seen in the Eastern AS during both seasons (Fig. 6c,d, with the positive trend extending to the Western AS during OND (Fig. 6c). As a result, Southeast AS is developing into a hot zone for the generation of severe cyclonic storms, as evidenced by the rising spatial trend in GPI over SEAS during both the MAMJ and OND seasons. An increasing trend is observed along the western coast, below ${15}^{\circ }\,\hbox {N}$ during MAMJ.

**Figure 7:**
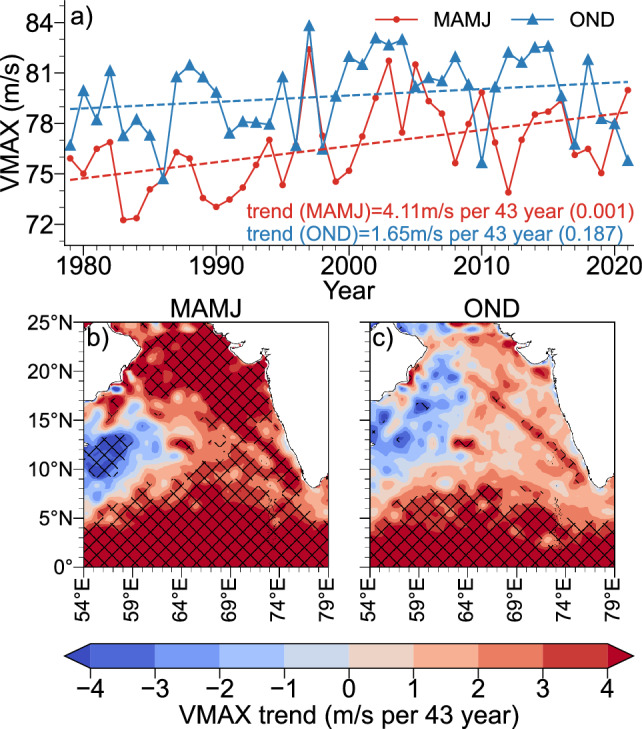
(**a**) Area-averaged time-series of VMAX (**b**, **c**) spatial trend of VMAX, for MAMJ and OND, respectively (the hatches indicate statistically significant areas at 95% confidence level).

From the analysis of spatial variability in the modified GPI, it is found that, large positive trend is observed and spread over entire EAS. The trend analysis of the time-series of area-averaged modified GPI also exhibit a large and significant positive trend during MAMJ and OND season as compared to GPI trend without including oceanic sub surface influence. This clearly signals the role of including ocean subsurface temperature in the GPI to comprehended the link of ocean subsurface in cyclogenesis under warming scenario. The potential intensity is the maximum steady intensity a storm can reach based on its energy cycle, where mechanical dissipation in the storm’s atmospheric boundary layer balances the heat input from ocean evaporation, multiplied by a thermodynamic efficiency^57^. The trend in the area averaged VMAX over EAS shown in Fig. 7a shows that, large significant trend in MAMJ season (${4.11}\,\hbox {m s}^{-1}$ per 43 years), which is almost three times the trend during OND season (${1.65}\,\hbox {m s}^{-1}$ per 43 years). As a result, the EAS region is becoming more favourable for the generation of intense cyclonic storms during the MAMJ season due to ocean warming and atmospheric instability. The spatial trend analysis of VAMX reveals a sizeable upward trend in EAS as well (Fig. 7b). The positive trend in VMAX during the OND season, however, is only present in the equatorial parts of the Arabian Sea (Fig. 7c). Despite the fact that local SST warming raises the VMAX as a result of global warming, there are several opposing factors at play, such as remote and local SST-induced circulation shifts in response to ENSO and IOD, where non-uniform subsurface warming mostly determines ocean subsurface stratification. Even while it is not spatially homogeneous and may be weak in some local areas and during OND season, the VMAX is nevertheless rising as a result of global warming. Because VMAX only takes into account surface temperature data, the ocean’s subsurface (changes in vertical structure) must also be carefully taken into account in addition to SST changes for TC genesis in a warming world.

**Figure 8:**
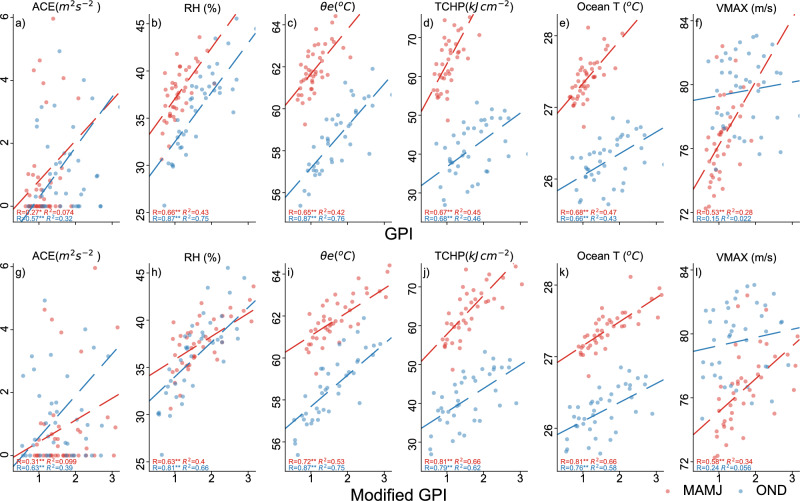
(**a**–**f**) Scatter plots and correlation of ACE, RH, $\theta _e$, TCHP, Ocean subsurface temperature and VMAX with GPI, respectively, (**g**–**l**) same as (**a**–**f**), but against modified GPI. Two stars (**) beside the **R** values indicate significance at 95% while a single star (*) indicate significance at 90% confidence level.

The correlation between seasonal mean subsurface water temperature and GPP during the peak TC season (March–June and October–December) from 1979 to 2021 over the Arabian Sea is examined and presented in Fig. 8e,k. This analysis consolidates the relationship of all influential atmospheric and oceanic thermodynamic parameters with GPI (Fig. 8). In general, most of the variables considered in this analysis exhibits strong positive and significant relationship with GPI except for VMAX during OND season. It is also evident that the relationship is getting stronger when we include the oceanic subsurface information in the modified GPI. It is also evident that, vertically averaged ocean subsurface temperature up to a depth of 150 m is showing slightly higher correlation values than TCHP correlation. Hence, importance of this ocean subsurface in cyclogenesis is also evident. The strong and significant correlation between VMAX and GPI during MAMJ season further supports our theory that the increase in cyclogenesis activity over EAS during MAMJ season is mostly caused by atmospheric instability and ocean surface temperature. However, during the OND season, there is no discernible relationship between VMAX and GPI. In contrast to MAMJ season, OND season shows a significant and high association between relative humidity and equivalent potential temperature and GPI. As a result, the vertical thermodynamical profile of the atmosphere and ocean has a complementing and occasionally opposing effect on cyclogenesis, which varies spatially and seasonally.

Furthermore, subsurface water temperature, which decides the vertical thermal structure of the ocean, may be responsible for the increase in frequency and intensity of TC, which is more evident in the spatial trend of modified GPI after including TCHP in the GPI. The strength of this effect depends on the ocean subsurface thermal structure. Thermodynamic state of the atmosphere and the ocean subsurface temperature profile over EAS and its link with seasonal GPI has been investigated. The subsurface ocean environment is not spatially uniform and the coupling between the subsurface ocean and atmosphere above may be distinct over different regions within Arabian Sea basin and increasing trends in the EAS both in original GPI and modified GPI confirms that, EAS is becoming a hot spot for cyclogenesis in recent decades.

### Discussion and conclusion

This study explores the influence of the changes in the vertical profiles of atmosphere and oceanic environment in clustering the cyclogenesis over EAS using observational estimates of thermodynamical profiles of the atmosphere and subsurface ocean during the period 1979–2021. This study also established the link between rising TC activity in the EAS and attempt to comprehend the underlying cause of the clustering and increasing TC activity in EAS during MAMJ and OND Seasons. According to the TC frequency and ACE analyses, there has been an increase in TC activity in the EAS since 1979. In the most recent eras, both seasons have seen a notable increase in the average number of cyclonic systems. The study found a notable rise in mid-tropospheric humidity during both seasons, with OND showing the largest rise. The recent epoch over the EAS during both seasons indicate a positive trend in the vertically averaged mid-tropospheric humidity, which is averaged between 800 and 300 hPa. Compared to MAMJ, the trend is noticeably stronger during OND and is primarily present in central and Southeast AS. According to the area-averaged vertical time-section of RH and the vertical profiles of its trend at each level, the mid-troposphere as a whole is seeing an increase in RH rather than just one specific level. In recent decades, SST has increased throughout the entire basin in both seasons.

Thermal instability, as shown by $\theta _e$, exhibits a similar growing trend, which is also observed throughout the OND season in all vertical levels over the EAS. The area-averaged time series of SST shows a positive trend. In other words, it should be highlighted that an increase in SST has been reported throughout the basin and that the rise in cyclogenesis potential over EAS is related to other oceanic patterns and processes. In the most recent epoch, TCHP has increased significantly, largely confined to northern parts during OND and in the northern and Southeastern regions of AS during MAMJ. This rise in TCHP at the eastern edge of the AS during MAMJ explains how TCHP contributes to the rising cyclogenesis potential in EAS. Given that the local SST is climatologically over the 26$^{\circ }$ threshold over this maritime region for the genesis and strengthening of TCs, the recent increase in TCHP suggests that the EAS is becoming increasingly favourable for TC activity. The increase in 26$^{\circ }$ isotherm depth and warming of the sub-surface water, both of which are positively contributing factors to the observed increase in TCHP during MAMJ, are evidenced by the trend towards higher sub-surface temperatures below 50 m.

The thermodynamic structure of the upper ocean and lower atmosphere have a significant impact on cyclogenesis over EAS. The main atmospheric regulating elements for the observed rise in cyclogenesis and its clustering in the eastern Arabian Sea are the increase in thermal instability and mid tropospheric humidity. By changing the vertical thermal structure of the ocean as a result of global warming, the subsurface oceanic processes favours the conducive environment for TC generation by extending thermal stratification over deeper layers in the ocean. In general, the relationship between ocean subsurface temperature and the TC activities indicated by GPP across the EAS was the main focus of this work. Simple correlation analysis is performed to describe the association between specific cyclogenesis parameters and GPP, which is a limitation of the procedure. Therefore further investigation is of great importance to evaluate the large-scale environment and governing physical mechanism behind the correlation in detail. Coupled model experiments is necessary to understand complex complementing and offsetting influences of ocean subsurface warming on the atmosphere in the cyclogenesis process. Accurate prediction of TC intensity in these cases probably requires accurate measurement of the upper-ocean thermal structure of the storm environment.

Given the population density on the western coast of the Indian peninsula, this study has important implications for forecasting, catastrophe risk reduction, and climate change adaptation. Along with enterprises, essential facilities, and infrastructure, there is a significant concentration of urban and rural habitats along the coast. Small-craft fishing operations and coastal environments are particularly susceptible to storm activity, making artisan fishing particularly vulnerable, especially along its southern boundaries. The report urges development strategies that account for the dangers posed by a changing climate and weather as well as policy and technological initiatives in the areas of storm warning, impact-based local weather services, and localised reliable weather services.

## Data and methodology

The study area encompasses the Eastern Arabian Sea (EAS), along the coast of the Indian peninsula, as depicted in Fig. 1a.

### Data

The frequency of cyclonic systems over the AS were obtained from the Cyclone e-Atlas provided by IMD^58^, while the cyclogenesis locations were obtained from Best track data from RSMC New Delhi. TC tracks from Joint Typhoon Warning Center (JTWC) Best-Tracks dataset^59^ was used for calculating the accumulated cyclone energy (ACE) as its computation require 6 hourly interval data of maximum sustained wind speed. The comparison of IMD and JTWC (figure not shown) shows that, both the independent data sets are in general agreement in terms of count, frequency, intensity and distribution of cyclones over Arabian Sea. The cyclone category used in this manuscript completely follows IMD criteria and we have considered only one category, ie tropical cyclone when maximum sustained wind speed exceeds 34kts. To maintain consistency, we have mainly used IMD data and JTWC is used only to supplement the analysis based on IMD best track data.

The monthly-mean atmospheric parameters and SST at a spatial resolution of ${0.25}^{\circ }\times {0.25}^{\circ }$ were taken from the ERA5 Reanalysis dataset released by ECMWF^60,61^. Depth of 26$^{\circ }$ isotherm (D26) and sub-surface temperature profiles were obtained from ECMWF’s Ocean ReAnalysis System 5 (ORAS5)^62^ to calculate TCHP.

For the trend analysis, linear least-squares regression was used, with a two-tailed Wald Test with t-distribution was used to calculate the *P* value.

### Methodology

The study period is restricted from 1979 to 2021 to exclude less-reliable data before the satellite-era and to include the recent data that is currently available. The climatology is calculated from 1979 to 2008 (30 years). Following sections describe the individual atmospheric and oceanic parameters, that have been used to analyse the relative contribution on the cyclogenesis potential during MAM and OND seasons. Area average of any parameter is taken over the area defined as EAS, which is highlighted in Fig. 1a.

#### Accumulated cyclone energy

The Accumulated Cyclone Energy (ACE) is a parameter indicative of the number, intensity and lifetime of tropical cyclones that form in a given time period^63,64^. ACE calculated by adding the square of 6 h MSW for all the systems while having an intensity of tropical cyclone or higher (${17.5}\,\hbox {m s}^{-1}$ or higher)^63^. ACE is calculated as^63^:

1 $$\begin{aligned} ACE=\sum V_{MSW}^{2} \end{aligned}$$

where $V_{MSW}$ is the maximum sustained wind in $\hbox {m s}^{-1}$.

#### Equivalent potential temperature ($\theta _e$)

Equivalent potential temperature ($\theta _e$) is a measure of convective available potential energy in the lower levels of the atmosphere, and has been used as a predictor for changes in TC intensity^47,48,65,66^. ($\theta _e$) is calculated using Eq. 2^67^:

2 $$\begin{aligned} \theta _e=\theta _{DL}\times exp\left[ \left( \frac{3036}{T_L}-1.78\right) \times r\left( 1+0.448r \right) \right] \end{aligned}$$

where $T_L$ is the temperature at the Lifting Condensation Level (LCL) $\theta _{DL}$ is the potential temperature at LCL. LCL is calculated using an iterative method in which the algorithm starts with finding the dewpoint from the LCL and starting mixing ratio followed by finding the LCL pressure from the starting temperature and dewpoint, and iterate until convergence^68^. $\theta _e$ was calculated using the Python library MetPy^68^.

#### Tropical cyclone heat potential

Tropical Cyclone Heat Potential (TCHP) is an important oceanic parameter that influences various stages of tropical cyclone life cycle^1,14,52,69–71^. It is defined as the integrated heat content from the sea surface to the depth of 26$^{\circ }$ isotherm (D26). TCHP is calculated using the following equation^1^:

3 $$\begin{aligned} TCHP=\rho \, C_{p}\int \limits _{0}^{D_{26}} (T-26) \, dz \end{aligned}$$

where $\rho$ is the average density of seawater, $C_{p}$ is the specific heat capacity of seawater at constant pressure, *T* is the temperature in degree Celsius at different levels of *dz* thickness, and $D_{26}$ is the depth of 26$^{\circ }$ isotherm.

#### Genesis potential index

The Genesis Potential Index (GPI)^44^ is a modified version of the TC Genesis Index by Gray^41^. GPI is defined as:

4 $$\begin{aligned} GPI=\left| 10^{5}\eta \right| ^{3/2}\left( \frac{RH}{50}\right) ^{3}\left( \frac{V_{pot}}{70}\right) ^{3}\left( 1+0.1 V_{shear}\right) ^{-2} \end{aligned}$$

where $\eta$ is the absolute vorticity at 850 hPa (in $\hbox {s}^{-1}$), *RH* is the relative humidity at 600 hPa (in percent), $V_{pot}$ is the potential intensity (in $\hbox {m s}^{-1}$), and $V_{shear}$ is the magnitude of the vector shear from 850 to ${200}\hbox {hPa}$ (in $\hbox {m s}^{-1}$).

Potential Intensity (PI) is the maximum theoretical threshold a storm could attain given the atmospheric and oceanic state^72–74^. Potential intensity, $V_{max}$, may be approximated by:

5 $$\begin{aligned} V_{max}^{2}=\frac{C_k}{C_D}\times \frac{(T_s-T_0)}{T_0}\times \left( h_{0}^*-h^*\right) \end{aligned}$$

where $C_k$ and $C_D$ are the enthalpy exchange and drag coefficients, respectively. $h_{0}^*$ is the saturation moist static energy at the sea surface and $h^*$ is the saturation moist static energy of the air above the boundary layer. $T_s$ is the ocean temperature, $T_0$ the mean outflow temperature^73^. The Potential Intensity is calculated using the Python module *pyPI*^75,76^. The GPI is further modified following^56^ by multiplying the GPI with TCHP scaled by 40. However, in order to account the spatial variability in the subsurface temperature and TCHP, spatially varying threshold based on climatology has been used to modify GPI in the present work.

### Supplementary Information

Supplementary Figures.

## Data Availability

Open access meteorological data used in this study are available from following websites: https://cds.climate.copernicus.eu (ERA5 and ORAS5 reanalysis data), http://14.139.191.203/AboutEAtlas.aspx (Cyclone eAtlas, IMD) and the JTWC best-track data from https://www.metoc.navy.mil/jtwc/jtwc.html?north-indian-ocean. Other environmental and contextual information will be available from the UK Data Service (UKDS, at https://ukdataservice.ac.uk) no later than May 2023. Access is subject to registration at the UKDS.
