# Supplementary material for: Changes in the thermodynamical profiles of the subsurface ocean and atmosphere induce cyclones to congregate over the Eastern Arabian Sea
Source: Sci Rep. 2023 Sep 22;13:15776. doi: 10.1038/s41598-023-42642-9 (PMC10516911; doi:10.1038/s41598-023-42642-9)
Supplement: Supplementary file 1 — Supplementary Figures. [file 41598_2023_42642_MOESM1_ESM.pdf]

Southeast Arabian Sea emerges as a hotspot for Severe Cyclonic Storms in a changing climate

Abhiram Nirmal C. S.[1], **Abhilash S.**[1, 2, *], **Max Martin**[1, 3, *], **Syam Sankar**[1, 4], **M. Mohapatra**[5], **and A. K. Sahai**[6]

[1]Advanced Centre for Atmospheric Radar Research (ACARR), Cochin University of Science and Technology (CUSAT), Kochi, 682022, India
[2]Department of Atmospheric Sciences, Cochin University of Science and Technology, Kochi, 682016, India
[3]Department of Anthropology, University of Sussex, Brighton, UK
[4]National Centre for Medium Range Weather Forecasting (NCMRWF), Sector-62, Noida, 201309, Uttar Pradesh, India
[5]India Meteorological Department (IMD), New Delhi, 110003, India
[6]Indian Institute of Tropical Meteorology (IITM), Pune, 411008, India

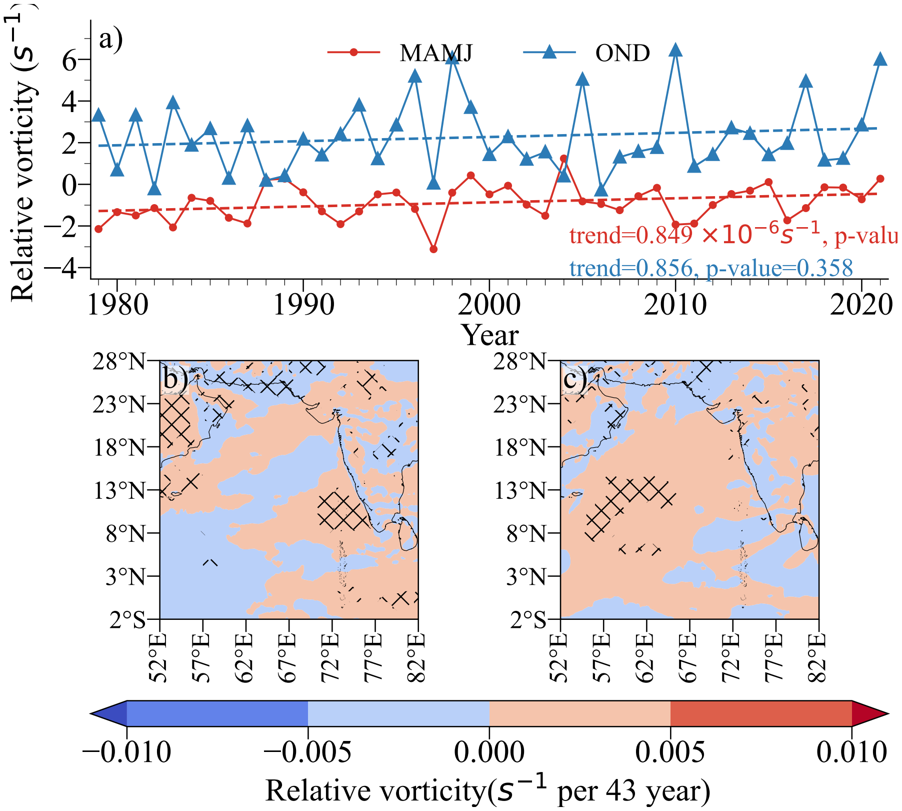

Supplementary Figure S1: a) Area-averaged relative vorticity and b&c) spatial trend of relative vorticity, for MAMJ and OND, respectively (the hatching indicates statically significant areas at 95 % confidence level)

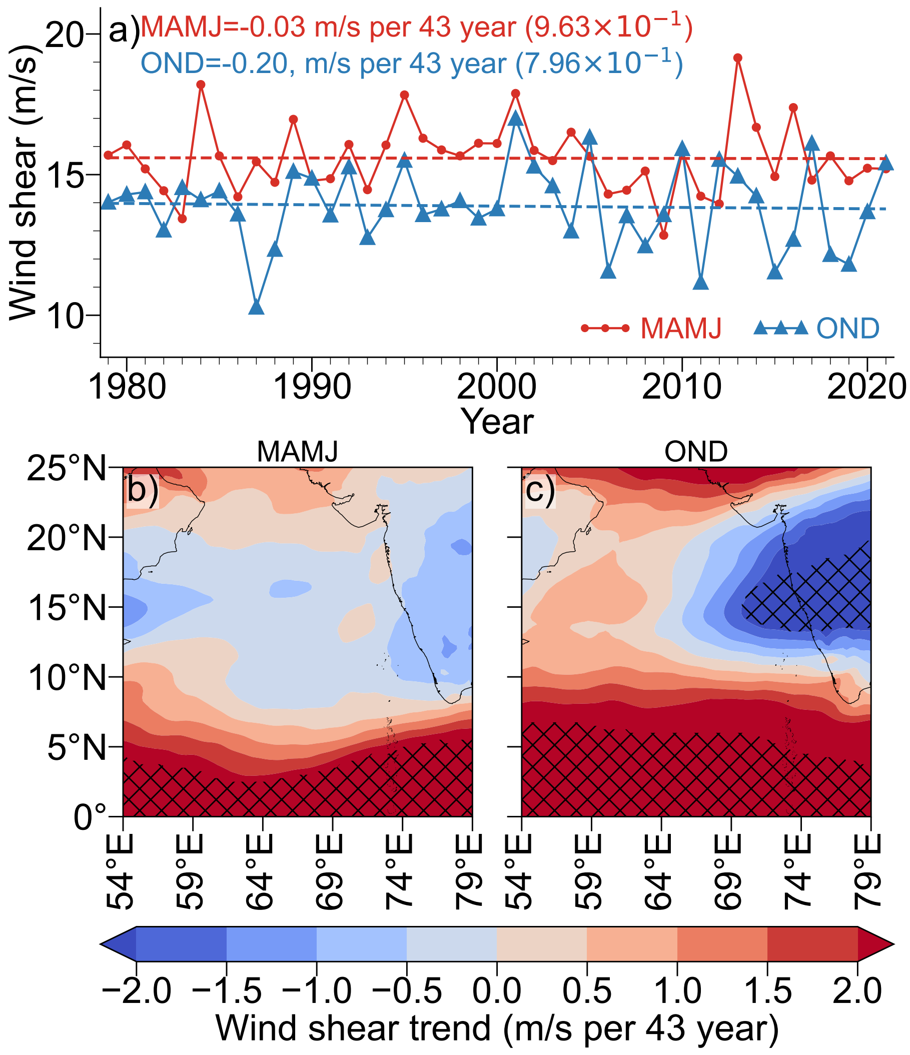

Supplementary Figure S2: a) Area-averaged wind shear b&c)spatial trend of wind shear for MAMJ and OND, respectively (hatching indicates statically significant areas at 95 % confidence level)

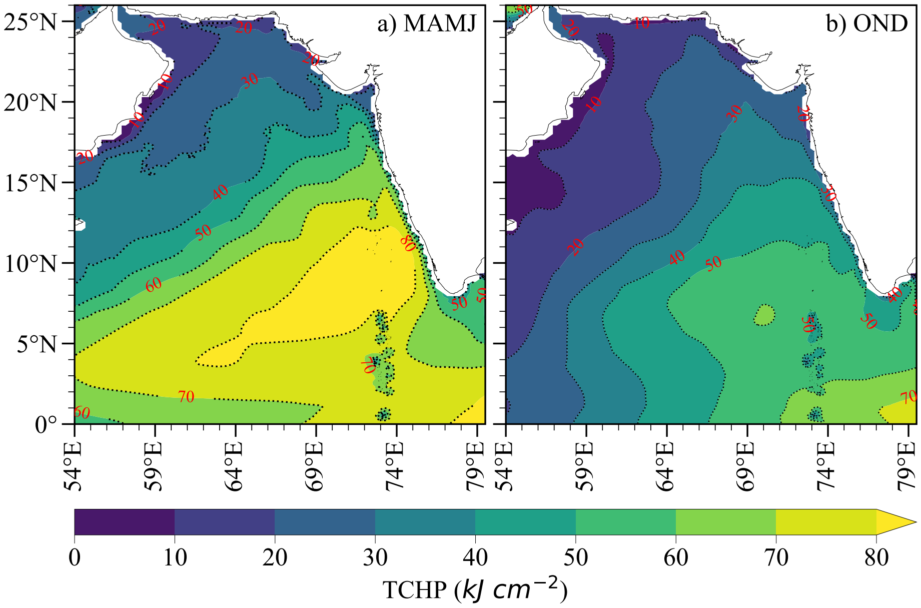

Supplementary Figure S3: TCHP climatology for a) MAMJ and b) OND
